# The Role of Footcare Nurses in Foot Lesions Treatment

**DOI:** 10.14789/jmj.JMJ21-0059-R

**Published:** 2022-06-02

**Authors:** YUKO TACHIBANA

**Affiliations:** 1 Juntendo University Hospital Podiatry-Center, Tokyo, Japan; 1 Juntendo University Hospital Podiatry-Center, Tokyo, Japan

**Keywords:** footcare, footcare nurse, foot lesions, evaluation, footcare team

## Abstract

Footcare awareness and practice are limited in Japan, which is attributable to unavailability of specialized podiatry services, in contrast to the Western healthcare system. Japan does not have national educational courses in podiatry and footcare, and daily foot care is not routinely practiced owing to the cultural background. Moreover, medical insurance covers only diabetic footcare, which contributes to the limited popularity of footcare in Japan. Footcare in Japan is provided by qualified nurses (foot care nurses) who are certified by various organizations and societies. Footcare nurses render the following services: (a) Provision of professional footcare after evaluation and patient education for foot self-care. (b) Multidisciplinary coordination between the footcare team. Owing to lack of podiatry services in Japan, a multidisciplinary therapeutic approach to foot lesions is necessary. The footcare nurse coordinates communication of patient information across team members and interdepartmental referrals for effective multidisciplinary therapy. (c) Patient education to improve awareness of footcare. Footcare is not currently widely established as a component of medical and nursing care and patient welfare, and greater awareness regarding its role is necessary. The importance of footcare to maintain healthy walking needs to be emphasized among individuals with foot lesions. In view of the high life expectancy and rapid population aging in Japan, maintaining a healthy gait is essential to improve healthy life expectancy, and foot care nurses can play an active role in the future.

## 1. Introduction

The term “footcare” was introduced in Japan only recently. In 2003, the Ministry of Health, Labor and Welfare added a “project for toe and nail care” to its long-term care prevention and community support program, and efforts were initiated to promote footcare in the domain of long-term care. The project aimed to reduce the risk of falls secondary to nail disorders through promotion of footcare among elderly individuals, their families, and care workers^1)^. In 2008, a new fee was included in the medical reimbursement system for the management of diabetic foot complications, which led to the implementation of footcare services in many hospitals. This fee included a medical fee for footcare rendered to patients with diabetes at high risk of foot lesions, and many medical centers/hospitals that were unable to provide footcare owing to lack of funds (medical fees for the service) established outpatient footcare clinics. However, the fees for management of diabetic complications can be calculated only for patients who meet the following criteria: (1) a history of foot ulcers and toe or lower limb amputation, (2) diagnosis of arteriosclerosis obliterans, or (3) diagnosis of diabetic neuropathy. Moreover, foot care services such as edema care, skin care, and nail clipping are not reimbursed and are often unprofitable with regard to revenue generation. In this context, many patients with non diabetic foot conditions including rheumatic deformities, hallux valgus, flat foot, foot paralysis associated with cerebrovascular disease, and ingrown toenails do not receive appropriate footcare at hospitals. Thus, since the start of the management of diabetic foot complications fee in 2008, foot care has become widely known in the field of diabetes treatment, but it is still not recognized in other fields.

Several individuals in Japan are unaware that their feet need care. Owing to the Japanese cultural milieu, footcare is not considered an important routine activity, in contrast to practices followed in Western countries. The shoe culture was established in Japan only over the past 100 years (compared with approximately 1,000 years ago in Europe and the United States); zori (sandals) and geta (wooden clogs) was the traditional footwear among the previous generations of the Japanese. Therefore, podiatry is not widely established as a medical speciality^2)^. Furthermore, culturally, footwear is considered a fashion accessory; shoes are often changed to match clothing, such as zori to match kimonos and shoes to match clothes. Usually, loose-fitting shoes such as zori and geta, which do not require the use of hands and are comfortable for the feet are preferred^3)^. Therefore, appropriate selection and correct use of shoes may not receive much attention, and shoe-induced chafing is treated as a common issue and calluses, chicken eye deformity, and ingrown toenails are ignored and remain untreated.

The lack of podiatrists and footcare specialists is an important contributor to the low levels of awareness regarding footcare in Japan. Podiatry is a separate and specialized field integral to medical practice in Europe and the United States. Podiatrists treat foot lesions with drug or surgical therapy similar to the role of dentists in Japan. Medizinischer Fusspfleger, which refers to a specialist in footcare and Shoe Meister, which refers to a specialist in shoes are national qualifications in this field in Germany. The Germans routinely visit a podiatrist for foot concerns, or visit a footcare salon on a daily basis and buy shoes under the guidance of a specialist. In contrast, in Japan, footcare is provided only by outpatient footcare clinics at some hospitals for patients with diabetes and at private footcare salons for cosmetic purposes.

The aging rate was 28.1% in Japan in 2018, and the mean life expectancy was 87 years for women and 81 years for men, which indicates significantly high longevity^4)^. Interestingly, that year, 6.44 million individuals were certified as requiring nursing care or support under long-term care insurance^5)^, and despite the long life expectancy, several Japanese are dependent on continuous medical and nursing care, and the gap between life expectancy and healthy life expectancy is concerning. It is important to maintain activities of daily living (ADL) and walking ability to extend healthy life expectancy^6)^. Maintenance of walking ability requires protection of foot health, which forms the primary focus of footcare. In essence, footcare plays a key role in preventive medicine and is indispensable to the rapidly aging Japanese society characterized by high longevity.

Juntendo University Hospital established the “Podiatry Center” as the first podiatry center in a university hospital in 2019, for comprehensive footcare services for all foot lesions regardless of the disease. The author is in charge of the outpatient footcare at this center. In this section, I will describe the role of footcare nurses in foot care, which is currently not well established in Japanese society.

## 2. Footcare nurses in Japan

As mentioned earlier, national qualifications for footcare specialists are not established in Japan; however, nurses with domain-specific qualifications recognized by various organizations and academic societies function as footcare nurses. Certifications for diabetic foot lesion specialization include the Certified Diabetic Nurse Practitioner certification provided by the Japan Nurses Association and the Japan Diabetes Care Instructor certification provided by the Japan Diabetes Care Instructor Certification Organization. Qualifications for specialization in foot ulcers and skin care include the Certified Nurse in Wound, Ostomy and Continence Nursing certification provided by the Japan Nurses Association. The Elastic Stocking and Compression Therapy Conductor certification provided by the Japanese Society of Phlebology, the Lymphedema Therapist certification provided by the Japanese Association for Lymphedema Therapy, and the Vascular Technician certification provided by the Vascular Technician Certification Organization are credentials for foot lesions of the vascular system. Qualifications that provide a wide range of knowledge regarding overall footcare include footcare instructors certified by the Japanese Society of Footcare and Podiatry and certification provided by the same society. Each of these qualifications trains professionals in a specific area of expertise; therefore, footcare nurses acquire multiple qualifications to provide comprehensive footcare and to compensate for the lack of knowledge and skills.

Qualified footcare nurses are engaged in foot care outpatient clinics at medical centers, dialysis clinics, and nursing homes^7, 8)^. However, not all qualified footcare nurses are engaged in footcare; there is a shortage of trained footcare nurses and medical centers and care facilities that provide foot care in Japan^9)^.

## 3. Role of footcare nurses

Footcare nurses provide the following services: (1) professional footcare, (2) management of the footcare team and, (3) patient education to improve awareness regarding the importance of footcare.

Implementation of footcare based on technical expertise and skills is important and includes not only direct patient care but also self-care and rehabilitation guidance and adjustment of the treatment environment.

In the absence of specialized podiatry services in Japan, a multidisciplinary team-based approach that includes a footcare team is necessary for management of foot disorders. Compared with other professionals, footcare nurses have more direct patient interaction and can better understand patients’ backgrounds and possible difficulties associated with treatment. Therefore, the footcare nurse is best suited for coordination with the multidisciplinary team to facilitate patient referrals for specialized treatment^10)^.

Despite the increasing need for footcare, medical, nursing, and welfare personnel in Japan are not particularly familiar with footcare services. Education of personnel involved in footcare is essential to provide appropriate footcare to patients with foot disorders. Moreover, the expression “footcare” is not widely recognized in Japanese society, and individuals with foot lesions tend to neglect these issues. Footcare nurses play a key role in education of medical, nursing, and welfare professionals regarding the importance of timely attention to foot lesions to maintain walking ability and for improvement of overall social awareness.

## 4. Evaluation and implementationof foot care

In this section, we will discuss evaluation necessary for the implementation of footcare and the contents of a footcare program.

### 4-1 Evaluation

#### 4-1-1 Foot Evaluation

In foot care, proper evaluation of the foot is very important. This chapter describes each item of foot evaluation.

##### 4-1-1-1 Lower extremity blood flow disorders

Patients with lifestyle-related diseases such as diabetes mellitus, dyslipidemia, hypertension, hyperuricemia, chronic kidney disease, obesity, and smoking should be considered high-risk patients for arteriosclerosis obliterans^11)^. During the early stages of lower extremity blood flow disorders, patients may experience only a sense of coldness; however, disease progression invariably manifests as intermittent claudication, and many patients seek medical attention for evaluation of this symptom. However, intermittent claudication also occurs in patients with lumbar spinal canal stenosis. Therefore, it is important to remember that patients with blood flow disorders may visit an orthopedic surgeon, and accurate diagnosis may be delayed. Patients with impaired blood flow through the lower extremities are susceptible to ulceration secondary to skin erosion and often develop intractable ulcers even after minor skin injuries. Additionally, severe lower extremity edema can lead to gangrene.

Physical examination should include measurement of skin temperature and color tone, palpation of the popliteal, posterior tibial, and dorsalis pedis, evaluation of hair loss, subjective symptoms, and the presence and extent of erosions and ulcers. Notably, friction may result in interdigital ulcers; therefore, careful foot inspection is necessary to ensure that no area is overlooked. Such evaluation can be performed merely through observation and palpation and does not require sophisticated equipment; such examinations can be performed by home healthcare and nursing home personnel. Measurement of blood flow using the ankle-brachial index, skin perfusion pressure, and magnetic resonance angiography, as well as cardiology consultation become necessary in cases of suspected or confirmed ischemia or ulcers.

##### 4-1-1-2 Neurological disorders

**Figure 1:**
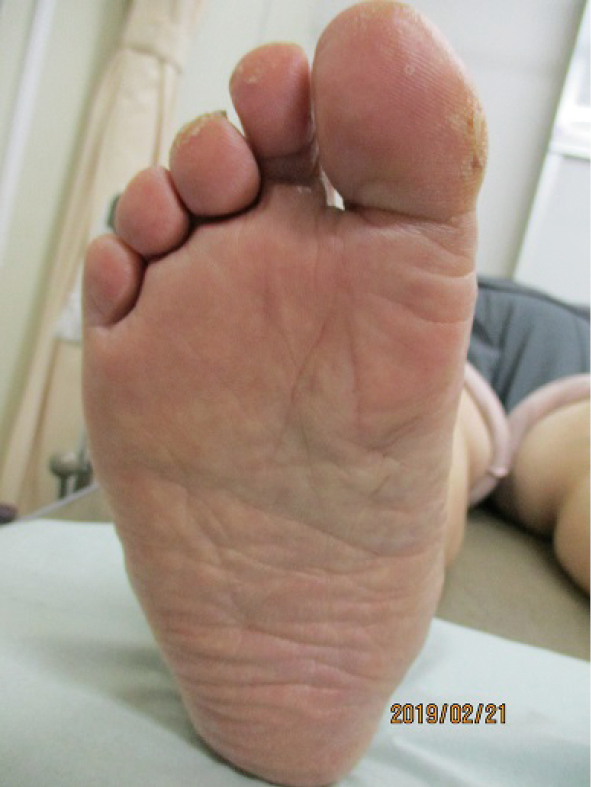
flat and open foot

**Figure 2:**
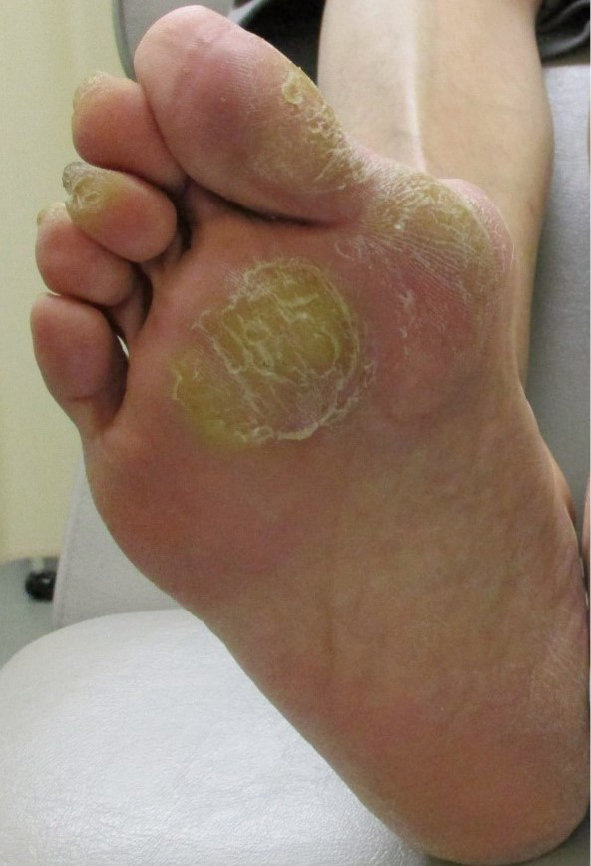
callus

**Figure 3:**
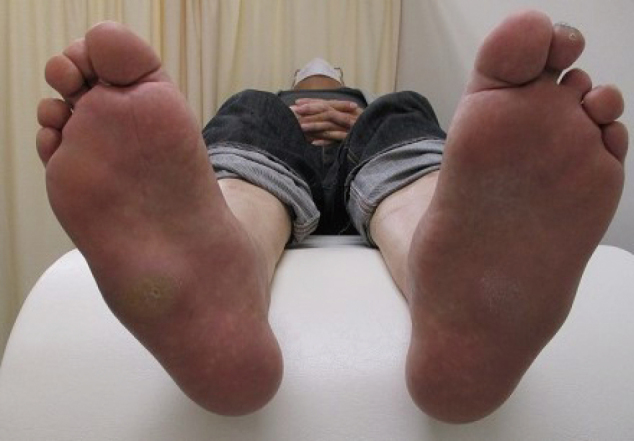
Charcot foot

Diabetic neuropathy, the most important among all peripheral neuropathies that affect the lower extremities is characterized by symmetrical appearance of the distal extremities^12)^. Sensory neuropathy results in decreased sensation of warmth and pain, which may delay detection of shoe-induced chafing and cold burns, with consequent serious foot lesions, which may even necessitate lower extremity amputation. Motor neuropathy causes muscle atrophy in the foot and joint contractures secondary to accumulation of advanced-glycation end products, which results in deformities such as hammertoes and crooked toes. Disruption of the arch structure leads to flat and open feet (Figure 1), with greater susceptibility to callus formation (Figure 2). Progressive autonomic neuropathy results in reduced sweating, which negatively affects the excretory function of the skin and causes dryness that predisposes patients to infection. Sympathetic neuropathy causes opening of arteriovenous shunts and consequently accelerated bone resorption and small fatigue fractures, which result in a characteristic Charcot foot deformity (Figure 3). Therefore, patients with diabetic neuropathy are at a high risk of multiple concomitant foot lesions.

In addition to diabetes mellitus, trauma, osteoarthritis, spinal disease, cranial neuropathies, prolonged alcohol consumption, and aging contribute to neuropathy. Similar to diabetic neuropathy, alcoholic neuropathy tends to show a distal and symmetric presentation; however, careful history taking and details regarding the patient’s lifestyle can distinguish between these conditions^13)^.

Evaluation of subjective symptoms, muscle atrophy, and the degree of deformity, if any, is important in addition to objective tests including the touch test, testing for vibration sense, and Achilles tendon reflex. It is also important to confirm shoe comfort and fit in patients with neuropathy because owing to sensory loss, patients are unable to determine the proper fit and tend to wear shoes that are too large or small.

##### 4-1-1-3 Edema

Lower extremity edema can be broadly categorized into generalized and localized types. Localized edema is mainly associated with foot lesions and includes lymphedema, venous edema, and disuse edema^14)^. In recent years, an increasing number of patients tend to present with disuse edema due to loss of muscle strength and reduced ADL^15)^. Medical treatment is prioritized in patients with systemic edema; however, lower extremity edema requires appropriate management under the guidance of a specialized department.

Edema-induced skin thinning causes dryness, a decline in the natural skin barrier function, and itchiness, which trigger scratching and predispose patients to infection after even minor wounds, resulting in cellulitis. Prolonged untreated edema can lead to fibrosis of the skin and loss of flexibility, which may result in joint contractures and motor dysfunction. Additionally, worsening edema can lead to intractable ulcers secondary to leakage of water that cannot be stored under the skin. Venous edema caused by varicose veins or impaired venous return typically manifests at the skin around the ankle joint, which shows pigmentation, leading to blistering, erosions, and stasis ulcers. Stasis ulcers produce a large amount of effusion and are painful and significantly negatively affect patients’ quality of life (QOL)^16)^. Use of elastic stockings is encouraged for management of lymphedema to avoid enlargement of the lower extremities and gait difficulties, which negatively affect QOL and ADLs^17)^. The degree of lower extremity edema varies with the duration of lower extremity weakness and the extent of activity. The foot size tends to fluctuate within the day, which can cause shoe-induced chafing, bedsores, and ingrown toenails. Furthermore, the increased weight of the lower extremity secondary to edema can cause gait difficulties and predispose the patient to falls.

Evaluation should be performed to determine the cause of the edema (systemic vs. localized). Screening for heart failure, liver and renal disease, hypothyroidism, and anemia is necessary to confirm systemic edema. Following exclusion of systemic edema, the cause of edema should be investigated using ultrasonography to measure venous return and, if necessary, lymphatic scintigraphy. Edematous feet may be scarred and show continuous subcutaneous fluid leakage. Even minor wounds may lead to cellulitis and should be closely monitored. Careful evaluation is necessary to determine patients’ range of motion and gait disturbances, if any, caused by skin fibrosis.

##### 4-1-1-4 Deformities

In addition to trauma- or fracture-induced deformities, foot deformities may be associated with osteoarthritis, rheumatoid arthritis, diabetic neuropathy, other peripheral neuropathies, aging, and use of inappropriate footwear. Foot deformities can lead to abnormal load balance during walking and cause calluses and clavus.

Metatarsalgia, an inward deformity of the first metatarsal bone is more commonly observed in women and is associated with rheumatoid arthritis, genetic factors, flat feet, weakness of the foot muscles, and inappropriate footwear^18)^. Progressive metatarsalgia is characterized by severely deformed and overlapping toes and causes difficulty with wearing shoes and walking. Hallux valgus, which is often mistaken for hallux valgus. Hallux valgus is characterized by callus formation on the dorsal aspect of the metatarsophalangeal joint of the first toe. Bunions tend to develop on the lateral aspect of the foot owing to footwear-induced friction, and a callus or clavus is often observed on the lateral aspect of the fifth toe. Hammertoe or claw-toe deformity is associated with diabetic neuropathy or inappropriate footwear, which results in calluses and clavus involving the proximal interphalangeal joint and tip of toes.

A flatfoot deformity is commonly observed in children and elderly individuals. Disruption of the arch structure, which causes a flatfoot deformity, is primarily caused by foot muscle weakness, with consequent plantar tendonitis and plantar fasciitis, which result in chronic pain and impaired walking ability^19, 20)^.

Charcot foot is characteristically associated with diabetic neuropathy (Figure 3) and occurs secondary to the accumulation of small fatigue fractures in the foot^21)^. Patients with diabetic Charcot foot are considered to have developed peripheral neuropathy, which clinically presents with reduced warmth and pain sensation, and ulcers resulting from calluses and shoe abrasions are often detected late and tend to become serious.

Evaluation includes inspection and palpation to confirm bony deformities, muscle and tendon atrophy, and joint range of motion. The patient’s load balance is evaluated in the erect position, and the extent of the deformity is confirmed radiographically. Callosities, clavus, and evidence of worn shoe soles are important indicators of load imbalance.

##### 4-1-1-5 Skin lesions

Evidence of skin dryness, desquamation, and maceration of the interdigital spaces is associated with a high index of clinical suspicion for tinea pedis. Tinea pedis is a common skin lesion that affects approximately 40% of the adult population^22)^ and is associated with hyperkeratosis, greater susceptibility to infection owing to skin dryness, aggravation of calluses, and fissuring. Lower extremity ischemia presents with reddish-purple discoloration and coldness of the skin over the ischemic areas. Alopecia and atrophy or loss of nails also indicate lower extremity ischemia. Patients who receive long-term steroid therapy show atrophic and fragile skin, and even mild irritation can cause ulcers.

Nail abnormalities include ingrown and thickened toenails, which are usually associated with inappropriately designed footwear, an incorrect fit or manner of wearing shoes, the patient’s gait, and toe deformities such as bunions, and onychomycosis. Nail abnormalities affect walking balance and predispose patients to falls^23)^.

Evaluation includes observation of skin thinning and fibrosis secondary to edema, skin atrophy secondary to chronic steroid medication, ischemic skin changes, redness secondary to deformities, evidence of calluses and clavus, and nail deformities. Microscopic examination should be performed for prompt diagnosis and treatment in cases of suspected toenail onychomycosis.

#### 4-1-2 Evaluation of general condition

##### 4-1-2-1 Factors that affect skin lesions

Patients who receive chronic steroid therapy often present with atrophic fragile skin that is vulnerable to injury even with mild irritation. Generalized or localized edema leads to skin thinning and easy scarring, which predispose to cellulitis. Dryness caused by diabetic neuropathy, edema, and aging is associated with itchiness, and scratching predisposes to infection secondary to impaired barrier function.

##### 4-1-2-2 Factors associated with reduced body defense mechanisms

Diseases associated with immune dysfunction, hyperglycemia, low nutrition, peripheral circulatory disorders, and aging affect the body’s defense mechanism; even minor wounds may get severely infected, healing is delayed and the skin is predisposed to ulcer formation and aggravation. Specifically, patients with diabetes and persistent hyperglycemia (blood glucose levels ≥250 mg/dL) show reduced ability to phagocytose neutrophils with greater susceptibility to infection.

##### 4-1-2-3 Factors associated with walking ability

Patients with cranial neuropathies, spinal disease, and osteoarthritis are susceptible to falls secondary to abnormal gait balance. Anemia and medication use (antipsychotics and anticancer drugs, among others) can also cause unsteadiness and increase the risk of falls. Patients with impaired lower extremity blood flow may develop intermittent claudication with difficulty in walking long distances. Gait imbalance secondary to a variety of factors may cause pain and joint deformity due to the load on the affected as well as the unaffected side.

##### 4-1-2-4 Factors that affect foot self-care

Daily self-care is a fundamental principle of effective foot care; however, several factors prevent foot self-care in patients. For example, hemiplegia due to cerebral infarction, motor dysfunction due to spinal disease or limited range of motion of joints, decreased visual acuity, decreased manual dexterity, decreased cognitive ability, and decreased adherence are known obstacles to self-care. Additionally, housing conditions without bathrooms, financial difficulties, and lack of support affect foot self-care. Patients tend to harbor misconceptions about footcare. For example, patients often treat calluses using scissors or clip nails sensitively, without direct visualization, which increases the risk of ulcer formation.

#### 4-1-3 Footwear evaluation

Careful scrutiny of footwear is often sufficient in patients with foot lesions; shoe width, size, shape, material, sole, heel, laces, and design should be closely examined. In addition to the shoes worn when visiting the outpatient clinic, it is necessary to examine the shoes that the patient uses frequently and also work footwear to confirm the effects of footwear on foot lesions^24)^.

Calluses and clavus on the feet unaccompanied by significant deformities are often indicative of footwear-induced lesions, which may be associated with an inappropriate size, high heels, and thin soles of shoes, as well as shoes with many designs and stitches at the back of the foot. Ingrown toenails, hallux valgus, and little toe varus are often caused by narrow-toed shoes, inappropriate shoe sizes, and high heels.

In addition to the type of footwear, the manner in which it is worn can result in foot lesions; shoes with loosely tied laces or those in which the foot is not well secured result in back and forth slippage of the foot and instability. Therefore, shoe-induced chafing, calluses, and clavus tend to occur more frequently, and toes are pushed into the shoe tip during the kicking motion of walking, which results in ingrown toenails.

Patients with diabetic neuropathy experience sensory loss and therefore prefer tighter shoes owing to the sensation that their footwear does not fit correctly. Poorly fitting footwear can cause shoe chafing; therefore, patients with diabetic neuropathy should undergo careful footwear evaluation^25)^.

### 4-2. Foot care

Footcare includes care of lesions such as calluses and nail treatment, as well as foot self-care guidance. Footcare should be prioritized based on the results of evaluation, and issues that require urgent attention should receive immediate care. Care is selected within the scope of self-care that can be implemented by the patient and family, and the method of implementation is taught. Patients and their families who cannot perform self-care should be referred to nursing care and welfare services. In this section, the specific content of foot care will be explained.

#### 4-2-1 Nail care

**Figure 4:**
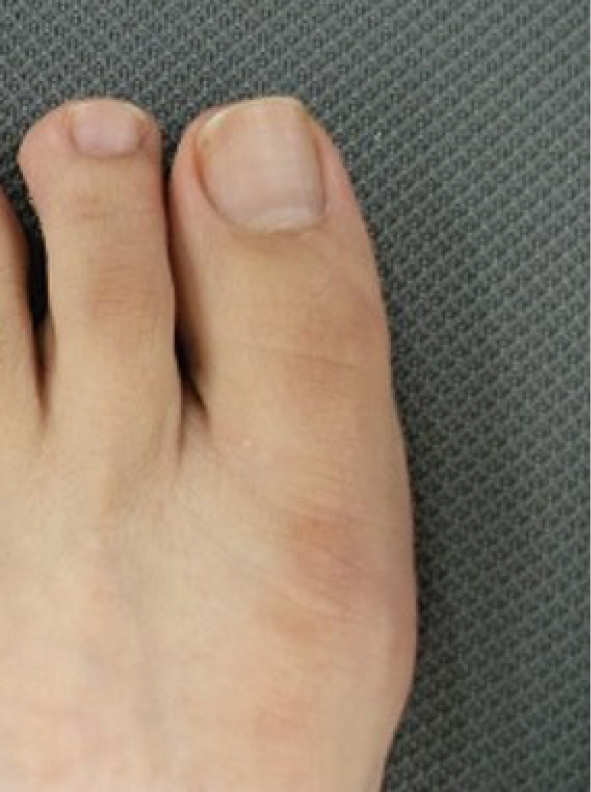
square-off

Nail clipping, trimming of thickened nails, and correction of ingrown nails are components of nail care. Usually, square-off nail trimming is recommended; the tip of the nail blade is cut straight and only the nail corners are rounded using a file (Figure 4). However, nails should be trimmed carefully to avoid skin injury considering the patient’s foot shape in those with toe deformities including bunions. Periungual inflammation may occur secondary to the accumulation of plaque around the nail in patients with thickened or ingrown nails^26)^. Taping and cotton packing may be useful for symptomatic treatment of ingrown nails and ingrown toenails; however, angle correction is necessary for pain or wounds. Ingrown nails and ingrown toenails often develop secondary to the shoe design and manner in which they are worn; therefore, it is necessary to provide guidance regarding the appropriate method of wearing shoes to prevent recurrence.

#### 4-2-2 Care of calluses and clavus deformities

Hardened, painful, bleeding calluses and clavus should be trimmed, and patients should be educated regarding factors that predispose to callus formation, such as foot deformities, shoe design, manner of wearing shoes, and an individual’s gait. The patient should also be instructed regarding daily cleansing and moisturizing care, because dryness causes friction and aggravates calluses. Alignment correction and decompression using plantar orthoses are effective for patients with calluses and clavus deformities. It is important to coordinate with prosthetists to improve these conditions. Patients who do not improve with outdoor foot orthoses alone require prostheses for indoor use as well . Taping and foot rehabilitation are also effective to prevent progression of foot deformities.

#### 4-2-3 Edema care

Compression therapy is effective for localized lower extremity edema; however, the degree, method (elastic stockings vs. bandages), and extent of compression should be considered on a patient-by-patient basis. Appropriate use of compression therapy depends on the patient’s motor and cognitive function; therefore, it is important to select a feasible method. Inappropriate compression therapy can worsen edema or cause pressure ulcers^27)^; therefore, it is important to perform compression therapy with the patient or family’s consent having confirmed that it can be performed appropriately. An edematous foot is prone to cellulitis; therefore, patients should be instructed regarding skin care, including cleansing and moisturizing. Lifestyle guidance including exercise therapy and weight loss may be necessary in patients with disuse- or obesity-induced edema. Manual lymphatic drainage massage is effective for lymphedema and venous stasis edema; a nurse trained in the appropriate technique performs the massage and the patient is accordingly instructed regarding the technique.

#### 4-2-4 Care of diabetic foot lesions

Footcare for patients with diabetes commences with evaluation of diabetic peripheral neuropathy. Owing to the progressive nature of this condition, it is difficult for patients to monitor changes in their feet in a timely manner. Therefore, patients with diabetes should have basic knowledge of preventive footcare from the early stage of diagnosis. Diabetic footcare does not include only management of peripheral neuropathy but also involves the treatment of several overlapping concerns such as impaired blood flow due to arteriosclerosis obliterans and reduced self-care ability due to retinopathy. It is important to establish a rapport between medical professionals and patients for timely advice regarding protection of their feet^28)^.

Progression of diabetic foot lesions is associated with glycemic control; persistent hyperglycemia is known to cause infection. Therefore, diabetic footcare must necessarily include close monitoring of blood glucose control and progression of complications other than foot lesions and the overall treatment of diabetes in addition to that of foot lesions.

#### 4-2-5 Care of footwear

It is well known that in addition to shoes with high heels and pointed toes, those with many seams and designs can cause foot lesions. Knowledge regarding selection of appropriate shoes and the correct methods of wearing them is limited among the Japanese; therefore, it is necessary to discuss these shoe-related footcare issues with patients. Footwear is a means of self-expression and individuality. Moreover, specific types of shoes may be designated by school or an individual's occupation; therefore, it may not be possible to change shoes from the viewpoint of “appropriate shoes for feet.” Therefore, it is necessary to provide shoes suitable for an individual and also offer education regarding the appropriate method of wearing shoes until the patient is convinced.

Correction of foot alignment is the fundamental principle of treatment of foot lesions. Creation of shoe insoles effectively relieves pain, treats calluses and clavus, and prevents progression of deformities. The Japanese medical insurance system provides coverage for orthotics once every 18 months.

## 5. Management of the foot care team

The footcare team includes orthopedics, plastic surgery, vascular surgery, cardiology, dermatology, diabetology, nephrology, rehabilitation, and anesthesiology (pain clinic), although these departments vary depending on the size of the medical institution. Nurses, clinical laboratory technicians, medical staff including physical therapists, exercise therapists, dietitians, pharmacists, and other prosthetists are also members of the footcare team. Therefore, establishment of a medical system comparable with podiatry services available in Europe and the United States warrants a multidisciplinary approach to improve the quality and efficiency of medical care. Compared with other professionals, footcare nurses have greater interaction with patients and are therefore better suited to understand patients' viewpoints and background. Footcare nurses provide footcare to patients across all stages and are capable of understanding a patient's condition closely, as the treatment phase shifts from the acute to the rehabilitation and subsequently the home treatment phase. Therefore, footcare nurses play an important role in multidisciplinary coordination, such as for communication of patient information across team members and interdepartmental treatment transfers. They also collaborate with those involved in nursing care insurance and home medical care to support patients' home care needs, to manage footcare within the range that can be implemented in the home environment, and to promote team medical care that includes those outside the hospital.

Therefore, several medical and nursing personnel from and outside hospitals participate in the footcare team. The footcare nurse plays a key role as a team coordinator, to support the transfer of treatment across various departments and professionals and for effective team functioning to ensure that patients receive the best and prompt medical care.

## 6. Role of foot care nurses in education and awareness activities

Western podiatry service are not available in Japan; therefore, the approach to foot lesions differs across medical centers/hospitals, which results in non-standardized medical care available to patients^29)^. Reportedly, lower extremity amputation is preferred over revascularization in patients with severely injured ischemic extremities, patients with deformed feet or nail abnormalities may remain untreated, and patients may develop pressure ulcers secondary to the use of thromboprophylactic elastic stockings^27)^. Currently, it is necessary to improve awareness among the Japanese involved in medical and nursing care and welfare in the field of limb salvage and footcare.

To address these concerns, the Japanese Society of Footcare and Podiatry has designated February 10 as Footcare Day and is actively involved in improving awareness regarding this important issue. Additionally, the society certifies footcare instructors who “aim to improve the footcare abilities (knowledge and skills) of patients and care providers and play a leading role in each field”^30, 31)^. Most footcare instructors are nurses who provide care in hospitals, as home health care aids, and home nursing care and also educate medical personnel, as well as patients and their families. Footcare nurses serve as models for footcare practitioners with their specialized knowledge and skills and are responsible for fostering footcare teams through their activities.

The establishment of an “additional fee for guidance and management of peripheral arterial disease of the lower extremities” in 2016 has focused attention on early identification of high-risk patients with lower extremity blood flow disorders^32)^. This medical fee enables referrals of high-risk patients with foot lesions to specialized hospitals. Footcare for ischemic extremities is not widely available in Japan currently; therefore, footcare nurses need to provide education to collaborating medical centers to ensure that the medical staff at the referral centers can continue patient care.

In Japan, owing to the cultural background, routine footcare is not widely recognized; therefore, public education is necessary to ensure that care of feet and their disorders are not neglected. It is important to improve awareness regarding the importance of footcare in the general population because even individuals without access to medical care include high-risk patients with foot lesions. In view of the increasing life expectancy and longevity trends in Japan, fall prevention and steps to prevent bedriddenness among the elderly population are important. Foot health also plays an important role in the national policy of extending healthy life expectancy. Optimal footcare can help individuals to regain their foot health, and those who receive footcare can serve as models of healthy longevity to improve awareness regarding footcare across the general population.

## 7. Conclusion

In this section, we discuss foot lesions associated with the maintenance of ambulation and the role of footcare nurses in the treatment of these disorders. Footcare is the mainstay of treatment for foot lesions and is necessary for all aspects of life, ranging from prevention to treatment and thereafter. In an aging society, the need for footcare will continue to increase. Therefore, there is an increasing need to train footcare nurses to play an active role in establishing a culture in which footcare is performed on a daily basis as an essential component of an individual's health care, similar to the practice followed in Western countries.

## Funding

No funding was received.

## Author contributions

YT contributed to the conception, drafting the paper, and preparing the figure.

## Conflict of interest statement

The Author declare that there are no conflicts of interest.
